# Promoter H3K4me3 and Gene Expression Involved in Systemic Metabolism Are Altered in Fetal Calf Liver of Nutrient-Restricted Dams

**DOI:** 10.3390/ijms26157540

**Published:** 2025-08-04

**Authors:** Susumu Muroya, Koichi Ojima, Saki Shimamoto, Takehito Sugasawa, Takafumi Gotoh

**Affiliations:** 1 Department of Animal Science and Welfare, Faculty of Veterinary Medicine, Kagoshima University, 1-21-24 Korimoto, Kagoshima 890-8580, Japan; 2 Division of Meat Animal and Poultry Research, NARO Institute of Livestock and Grassland Science, 2 Ikenodai, Tsukuba 305-0901, Ibaraki, Japan; 3 Laboratory of Sports Medicine, Department of Clinical Medicine, Institute of Medicine, University of Tsukuba, 1-1-1 Tennodai, Tsukuba 305-8572, Ibaraki, Japan; 4 Field Science Center for Northern Biosphere, Hokkaido University, N11W10 Kita, Sapporo 060-0811, Hokkaido, Japan

**Keywords:** epigenetics, fetal growth restriction, histone modification, liver, maternal undernutrition, metabolic programming, integrated stress response

## Abstract

Maternal undernutrition (MUN) causes severe metabolic disruption in the offspring of mammals. Here we determined the role of histone modification in hepatic gene expression in late-gestation fetuses of nutritionally restricted cows, an established model using low-nutrition (LN) and high-nutrition (HN) conditions. The chromatin immunoprecipitation sequencing results show that genes with an altered trimethylation of histone 3 lysine 4 (H3K4me3) are associated with cortisol synthesis and secretion, the PPAR signaling pathway, and aldosterone synthesis and secretion. Genes with the H3K27me3 alteration were associated with glutamatergic synapse and gastric acid secretion. Compared to HN fetuses, promoter H3K4me3 levels in LN fetuses were higher in *GDF15*, *IRF2BP2*, *PPP1R3B*, and *QRFPR* but lower in *ANGPTL4* and *APOA5*. Intriguingly, genes with the greatest expression changes (>1.5-fold) exhibited the anticipated up-/downregulation from elevated or reduced H3K4me3 levels; however, a significant relationship was not observed between promoter CpG methylation or H3K27me3 and the gene set with the greatest expression changes. Furthermore, the stress response genes *EIF2A*, *ATF4*, *DDIT3*, and *TRIB3* were upregulated in the MUN fetal liver, suggesting involvement of the response in *GDF15* activation. Thus, H3K4me3 likely plays a crucial role in MUN-induced physiological adaptation, altering the hepatic gene expression responsible for the integrated stress response and systemic energy metabolism, especially circulating lipoprotein lipase regulation.

## 1. Introduction

The feeding strategy is essential for producing beef cattle with high performance and efficiency. Low-quality management of beef cattle with under-/overnutrition of amino acids (AAs), fatty acids (FAs), proteins, vitamins, and/or minerals leads to poor meat yield and quality [1]. Particularly in the early developmental stages, the environment and nutrition have a serious impact on the health of individual animals in later life [2,3]. Proteins, AAs, vitamins, and minerals have been intensively studied as crucial nutrients affecting offspring metabolism during early life [3,4]. Evidence from epidemiological and experimental studies has indicated that malnutrition in fetal and infant animals predisposes their systemic metabolism and homeostasis to chronic disorders in adulthood [5], which potentially has transgenerational adverse effects on the life-long health of their offspring [6].

In the case of maternal undernutrition (MUN) in utero, the liver, skeletal muscles, and adipose tissue in fetuses are seriously impaired in their growth, leading to fetal (intrauterine) growth restriction (FGR; IUGR) [7,8]. The nutritional levels of AAs and methyl donor metabolites are particularly important in metabolic programming phenomena, as shown by their high impact on metabolic diseases in later life and offspring [9,10,11]. From another point of view, over-/undernutrition conditions in the early developmental stage have the potential to alter systemic metabolism enabling individuals to gain further accumulation of intramuscular fat (IMF) in Japanese Black cattle, a renowned breed for its highly marbled beef.

Epigenetic markers have been proposed as the molecular mechanisms underlying metabolic programming. In this context, prolonged nutritional stress alters DNA methylation, histone protein modification, and noncoding RNA expression in tissues that are crucial for systemic metabolism. In particular, DNA methylation at the CpG island in the gene promoter region suppresses gene transcription, whereas the effect of histone modification on gene expression varies, mainly depending on the modification site of histone 3 and the type of modification such as methylation and acetylation [12]. The epigenetic changes lead to changes in metabolic gene activation and protein activity [5,12,13,14,15], leading to phenotypic changes, even in the fetus, under maternal malnutrition. Fetal liver development, growth, and function are susceptible to restricted nutritional levels or calories in pregnant mice [16], rats, guinea pigs, sheep, and cattle [17,18,19,20,21]. In the rat liver, maternal protein restriction during gestation altered CpG dinucleotide methylation in the promoter of *GR* [22] and *PPARA* [23].

Recently, we investigated the effect of MUN on metabolomic and transcriptomic profiles in fetal calf liver and skeletal muscle using cows fed a low-nutrient diet (LN) in terms of carbohydrates, proteins, and lipids [21,24]. The MUN altered metabolite contents including AAs and gene expression associated with the suppression of tissue growth and functional maturation, toward thrifty energy homeostasis and tissue growth retardation. These results are consistent with the small tissue phenotypes of the LN fetuses [25]. In subsequent reduced representation bisulfite sequencing (RRBS) analysis of genome-wide DNA methylation in the fetal liver, promoter methylation of genes such as *POR*, *GNAS*, and *GRB10* was highly different between the LN and high-nutrient (HN) control groups [26]. However, these differentially methylated genes (DMGs) were not the major genes exhibiting marked expression differences between the LN and HN groups. In contrast, some of the genes such as *QRFPR* did not show significant methylation differences between the groups but gene expression significantly increased in the LN with a great difference compared to the HN [21,26]. This result led us to hypothesize that the majority of the markedly changed gene expression in the MUN fetal liver was modulated by histone modification. However, the impact of MUN on histone modification, still less the roles of histone modification in MUN fetuses and offspring, has been poorly investigated. Nevertheless, in an epidemiological study, histone 3 lysine 4 trimethylation (H3K4me3) unfolding was observed in the blood cells in children and mothers living in an urban slum in Bangladesh, particularly as redistribution of metabolic and immune genes [27]. Furthermore, maternal protein restriction (MPR) caused DNA hypomethylation and increased H3K4me3 of the hepatic *G6PC* promoter in piglets [28]. In sheep, restriction of concentrate in the maternal diet led to decreased histone 3 lysine 27 trimethylation (H3K27me3) of the hypothalamic *GR* promoter in the offspring [29].

To address the vailed roles of histone modification in MUN fetuses, here we conducted genome-wide chromatin immunoprecipitation sequencing (ChIP-seq) analyses focusing on the epigenetic role of H3K4me3 and H3K27me3 in gene expression in the fetal calf liver, comparing the roles of DNA methylation. Dams of Japanese Black (JB) cattle were fed LN and HN at nutritional requirement levels based on protein, fat, and energy content during gestation [21,24,25], which has been demonstrated that the present design provided a significant phenotypic difference in a variety of tissues between LN and HN fetuses. Based on the established condition that has been discussed previously [21,24,25], we consider the HN treatment to be an appropriate option as a control.

## 2. Results

### 2.1. Phenotypic Effect of MUN on Fetuses

We previously confirmed the effect of MUN on fetal growth and liver weight at 8.5 months of gestation. BW and liver weight (LW) were significantly lower in LN than in HN fetuses (Table 1). The LN/HN ratios of BW and LW were 0.74 and 0.79, respectively. Meanwhile, the LW/BW ratio did not differ between the LN and HN fetuses. Thus, the effect of MUN on the liver is proportional to and linked to systemic body metabolism.

### 2.2. Distribution of MUN-Induced H3K4me3 and H3K27me3 Mark Changes

**Figure 1:**
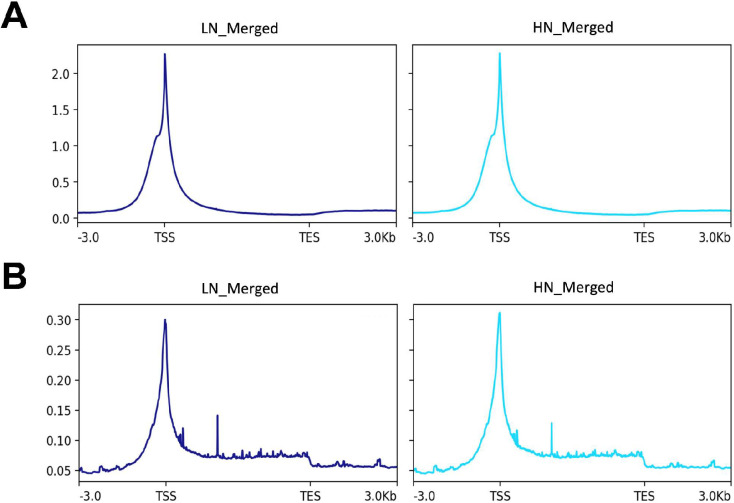
Distribution of histone marks in a region from −3 kb from the transcription starting site (TSS) to +3 kb from the transcriptional ending site (TES). (**A**); H3K4me3, (**B**); H3K27me3. LN; low nutrition, HN; high nutrition. The input sample of pooled LN and HN samples is used as a control.

Since H3K4me3 and H3K27me3 play crucial roles in transcriptional regulation, particularly at loci near the transcription starting site (TSS), we first analyzed the distribution of histone marks within a range of −3 kb from the TSS in the gene structure. The results showed a single sharp peak of the modification mark at the TSS of genes for both H3K4me3 and H3K27me3 (Figure 1). In addition, weak H3K27me3 signals spread broadly within the gene structure from the TSS to the TES, whereas no such signal was observed for H3K4me3 in the region. No apparent difference in overall histone mark distribution was observed between LN and HN fetuses.

**Figure 2:**
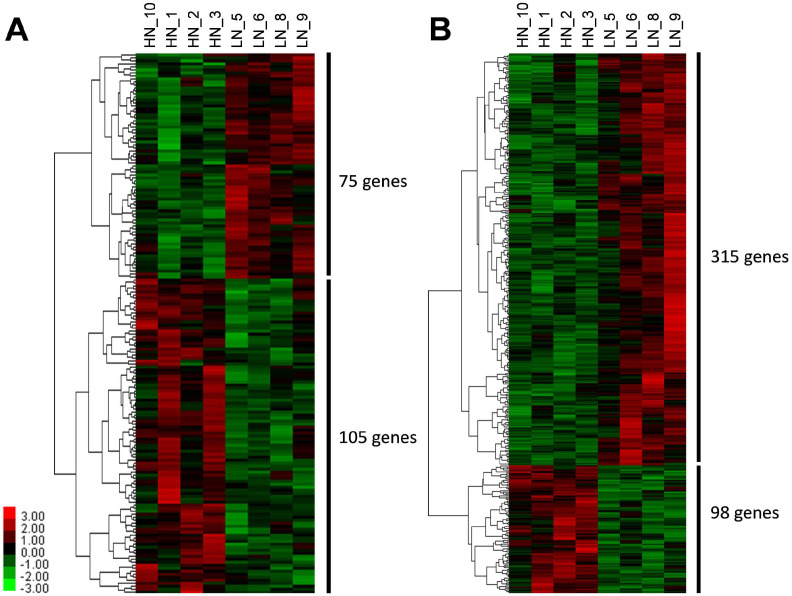
Hierarchical clustering analysis results of differentially histone-modified genes (DHG). DHGs were extracted by difference at *p* < 0.01. (**A**) H3K4me3, (**B**) H3K27me3. LN; low nutrition, HN; high nutrition.

Next, we explored differentially modified regions (DHRs) with H3K4me3 or H3K27me3 marks such that at least one peak end was adjacent to the TSS of a gene. The present study detected 20,823 H3K4me3 and 35,363 H3K27me3 regions in the fetal calf liver genome (Table 2). Of these, 535 H3K4me3 DHRs were significantly upregulated, and 404 DHRs were downregulated by MUN in the LN fetal liver (*p* < 0.05). In the case of H3K27me3, 1934 DHRs were upregulated and 1909 DHRs were downregulated in LN fetuses (*p* < 0.05). Furthermore, to extract genes transcriptionally regulated by H3K4me3 and/or H3K27me3, we focused on loci ranging from −1 kb to +1 kb from TSS as a functional region for transcriptional regulation. The distribution of genes with significantly changed histone marks is exhibited as heatmap results for hierarchical clustering analysis (HCA) (Figure 2), showing that 75 and 105 genes showed hyper and hypo H3K4me3 modification, respectively, at around TSS in LN fetuses. Meanwhile, 315 and 98 genes showed hyper and hypo H3K27me3 modification, respectively. Accordingly, LN and HN fetal liver samples were distinguished based on the distribution of the altered histone marks of these genes.

### 2.3. Genes and Metabolisms/Pathways Relevant to H3K4me3 and H3K27me3 Alteration

As summarized in Table 3, the genes exhibiting the greatest changes in H3K4me3 and H3K27me3 are crucial for hepatic lipid metabolism (*RARB*, *FADS1*, and *LDLRAP1*), gluconeogenesis (*CLK2*), neutral amino acid transport in fetuses (*SLC38A4*), and the immune system (*MIC1*, *UAP1*, and *GFI1*).

Further, gene ontology (GO) and Kyoto Encyclopedia of Genes and Genomes (KEGG) pathway analyses were performed to understand the metabolisms/pathways potentially regulated by MUN-associated histone modification, using differentially histone-modified genes (DHGs). The results of GO analysis indicated that the H3K4me3 plays a regulatory role in biological events, such as the regulation of transcription, apoptotic process, integrin-mediated cell adhesion, cell migration, cellular response to corticotropin-releasing hormone and insulin stimulus, and endothelial cell chemotaxis (Table 4). Meanwhile, according to KEGG pathway analyses, genes with H3K4me3 alteration were associated with the synthesis and secretion of cortisol, aldosterone, parathyroid, Cushing syndrome, PPAR signaling pathway, and cholinergic synapse.

In the analyses of genes with H3K27me3 alteration, the extracted GO terms were associated with biological processes including regulation of transcription, neuron differentiation and migration, developmental pattern specification, embryonic development, and cell differentiation (Table 5). Additionally, the calcium signaling pathway, Wnt signaling pathway, Rap1 signaling pathway, glutamatergic synapse, and GABAergic synapse were predicted to be H3K27me3-linked by KEGG pathway analysis. These results indicate that the potential regulatory roles of H3K4me3 and H3K27me3 in biological processes and pathways are different in the MUN fetal calf liver.

### 2.4. Difference in Epigenetic Modification and Altered Gene Expression Between Histone Methylation and DNA Methylation

It is widely accepted that H3K4me3 around TSS contributes to the transcriptional activation of gene expression, whereas CpG methylation or H3K27me3 alone suppresses gene expression [30]. However, little is known about which epigenetic factors have the greatest impact on altered gene expression in MUN fetal liver genes. We hypothesized that each epigenetic factor plays a distinct role in fetal liver gene regulation under MUN exposure. Based on this notion, to evaluate the potential performance of transcriptional modulation by H3K4me3 and H3K27me3, we compared the gene expression profiles linked with the epigenetic modifiers H3K4me3, H3K27me3, and CpG methylation using the top 20 differentially histone- or promoter CpG methylation-modified genes (DHGs or DMGs) in terms of two points: (1) whether each of the top 20 genes under each epigenetic modifier was theoretically upregulated or downregulated and (2) how frequently and greatly each epigenetic modifier transcriptionally altered the top 20 genes. To address this, we used the LN/HN ratio for epigenetic modification and gene expression as indices of epigenetic impact on altered gene expression.

**Figure 3:**
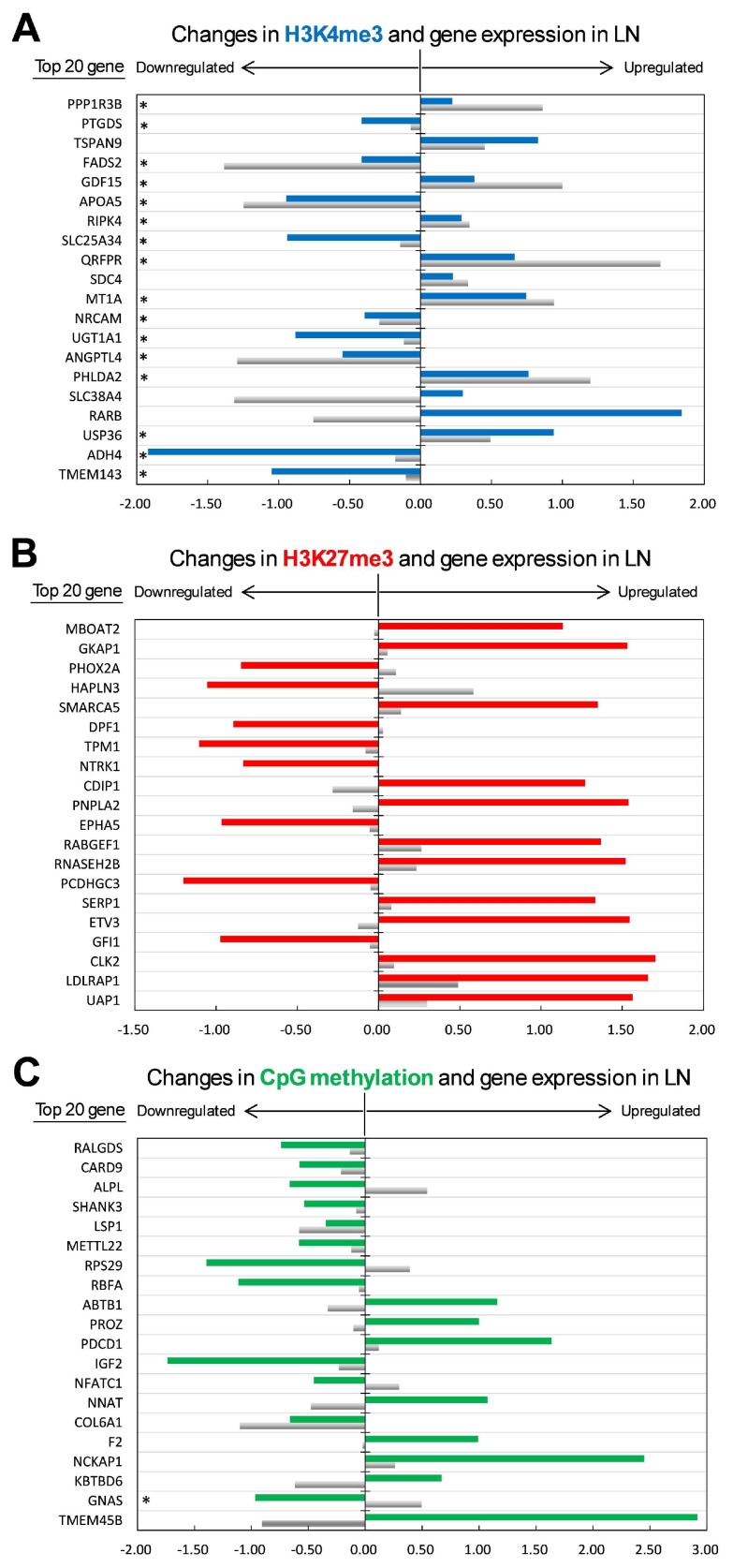
Difference in LN/HN ratio of epigenetic modification and gene expression between epigenetic modifications ((**A**): H3K4me3, (**B**): H3K27me3, (**C**): CpG methylation). Regulatory impact on gene expression was analyzed using the top 20 genes for each modification. Theoretical consistence: genes are marked with * if satisfying (1) a significant difference in expression between LN and HN at *p* < 0.05 and (2) altered expression toward the predicted up/down direction by the epigenetic modification. It is generally accepted that increasing the promoter H3K4me3 level activates gene expression, meaning that changes in H3K4me3 and gene expression are positively correlated. Meanwhile, increasing promoter H3K27me3 or CpG methylation suppresses gene expression, meaning that changes in the epigenetic modification and gene expression by MUN are negatively correlated. Horizontal and vertical axes indicate levels of HN/LN ratio (log2(LN/HN) value) and epigenetically changed genes, respectively. The bar color blue, H3K4me3; red, H3K27me3; green, CpG methylation; gray, gene expression.

Notably, of the top 20 DHGs in the H3K4me3 (*p* < 0.0016), 16 genes were significantly altered (*p* < 0.05), and the remaining four genes showed an altered expression trend (*p* < 0.10) in their expression (Figure 3A). Expression of the representative target genes, *ANGPTL4*, *MT1A*, *QRFPR*, *APOA5*, and *FADS2* was validated by qPCR (*p* < 0.05). In addition, the regulation of the 16 genes with significantly altered expression was theoretically consistent with upward or downward regulation by H3K4me3. Furthermore, the expression of 10 genes (*RARB*, *SLC38A4*, *PHLDA2*, *ANGPTL4*, *MT1A*, *QRFPR*, *APOA5*, *GDF15*, *FADS2*, and *PPP1R3B*) in LN fetuses was changed >1.5-fold compared to HN fetuses. Thus, as shown in Figure 3A, 16 genes of the top 20 H3K4me3-changed genes exhibited significant upregulation in LN when H3K4me3 modification increased, or significant downregulation in LN when H3K4me3 modification decreased.

In contrast, seven of the top 20 DHGs in the H3K27me3 modification (*ETV3*, *PNPLA2*, *CDIP1*, *DPF1*, *HAPLN3*, *PHOX2A*, and *MBOAT2*; *p* < 0.001) exhibited theoretically consistent alterations in gene expression; however, none of the expression was significantly different between LN and HN fetuses (*p* > 0.05; Figure 3B). In addition, no significant > 1.5-FC difference in gene expression was observed among the top 20 genes with H3K27me3. Similarly, ten DMGs with promoter CpG methylation (*TMEM45B*, *GNAS*, *KBTBD6*, *F2*, *NNAT*, *NFATC1*, *PROZ*, *ABTB1*, *RPS29*, and *ALPL*; *p* < 0.001) exhibited consistent alterations in gene expression (Figure 3C). However, only *GNAS* expression was significantly altered by CpG methylation (*p* < 0.05). *LSP1* was the only gene with a significantly altered expression of >1.5 FC (*p* < 0.05), although the expression alteration was not consistent with promoter CpG methylation. These results indicate that H3K4me3 is a major player in altering gene expression in the nutritionally restricted fetal liver, possibly via transcriptional regulation.

### 2.5. Relationship Between H3K4me3 and Gene Expression in MUN Fetal Liver

**Figure 4:**
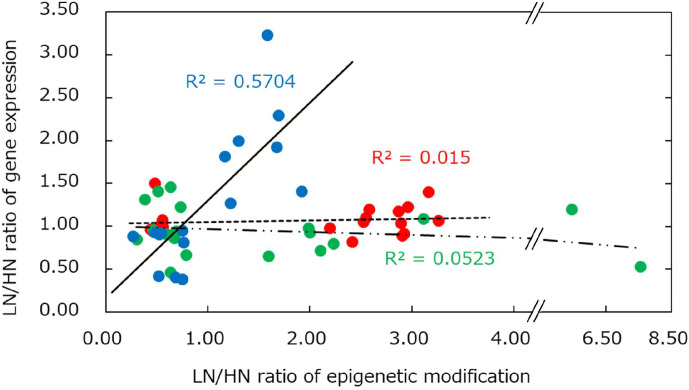
Relationships between changes in epigenetic modification and gene expression levels. Horizontal and vertical axes indicate the epigenetic modification ratio of low nutrition to high nutrition (LN/HN) and that of gene expression, respectively. The top 20 highly modified genes for each epigenetic modifier are plotted (see Figure 3). Regarding H3K4me3, genes without significantly altered gene expression (*p ≥* 0.05) are not plotted. Blue; H3K4me3 (—), red; H3K27me3 (---) green; CpG methylation (− ∙ ∙).

We further investigated the effect of H3K4me3 on gene expression in the MUN fetal liver. The correlation between H3K4me3 modification and the gene expression ratio of the consistently regulated 16 genes is illustrated in Figure 4. A positive correlation was observed between H3K4me3 and significantly altered gene expression (*R*^2^ = 0.5704), indicating a positive regulatory role for H3K4me3 in activating these genes in the MUN fetal liver. In particular, the genes altered in the expression by H3K4me3 are essential in hepatic metabolism and stress response, such as lipid metabolism regulators *ANGPTL4* and *APOA5*, a cellular glucose homeostasis regulator *PPP1R3B*, a crucial enzyme catalyzing glucuronidation *UGT1A1*, and a major cytokine in the integrated stress response (ISR) *GDF15*.

**Figure 5:**
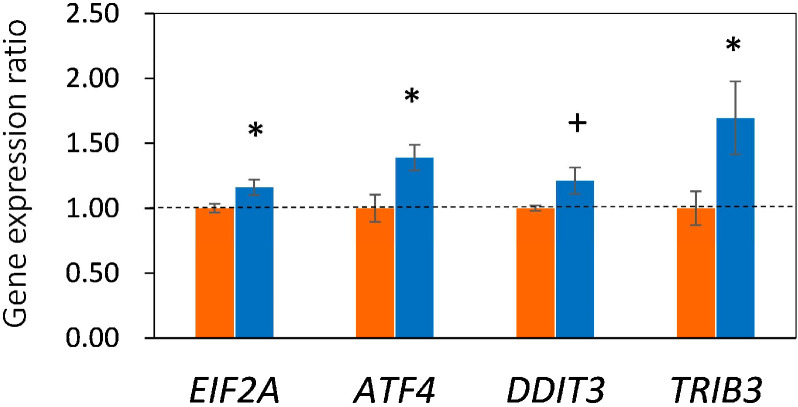
Gene expression ratio of low nutrition to high nutrition (LN/HN) of *EIF2A*, *ATF4*, *DDIT3,* and *TRIB3*. * and + indicate difference at *p* < 0.05 and *p* < 0.10, respectively. HN, orange, LN; blue.

The altered H3K4me3 and expression of *GDF15* suggested that endoplasmic reticulum (ER) and mitochondrial stresses induced by MUN led to ISR through the ISR-ATF4 pathway, which comprises crucial transcription factors such as *ATF4*, *EIF2A*, *DDIT3*, and *TRIB3*. Accordingly, we further examined the expression of these ISR-related genes. We found that *ATF4*, *EIF2A*, *DDIT3*, and *TRIB3* were activated in LN fetuses compared to HN fetuses (*p* = 0.021, 0.035, 0.055, and 0.041, respectively; Figure 5), in parallel with elevated *GDF15* expression. Thus, the results indicated that the LN fetal liver was in a physiological adaptation process in response to MUN.

## 3. Discussion

### 3.1. Potential Roles of H3K4me3 and H3K27me3 in Gene Expression of MUN Fetal Calf Liver

Our present results, targeting the effect of MUN on fetal calf liver, revealed distinct roles of H3K4me3 in altered metabolic gene expression during late gestation. This is the first study to analyze genome-wide histone modifications in the livers of ruminant fetuses treated with MUN. Alteration of histone modifications in MUN offspring has been observed in several previous studies including studies focusing on the livers of rats and piglets [22,31,32,33,34].

Here, we identified 105 upregulated and 75 downregulated genes in H3K4me3 and 98 upregulated and 315 downregulated genes in H3K27me3 in the MUN fetal liver. H3K4me3 and H3K27me3 have distinct functions in the regulation of gene expression. H3K4me3 primarily activates transcription, whereas H3K27me3 suppresses transcription, particularly in the early developmental stage, with an exceptional role in bivalent modification [30]. Our study observed differences in the genes with the greatest changes in H3K4me3 and H3K27me3 (Figure 3) and the associated metabolisms and pathways (Table 4 and Table 5). Genes with elevated H3K4me3 levels were associated with regulating transcription, apoptosis, and response to corticotropin and insulin as representative molecular events. In contrast, those with reduced H3K4me3 levels were associated with the synthesis/secretion of cortisol and aldosterone, Cushing syndrome, and the PPAR signaling pathway (Table 4). Regarding H3K27me3, the upregulated modification was associated with transcriptional regulation and neuronal differentiation and migration, whereas the downregulated modification was associated with the Ca^2+^, Wnt, and Pap1 signaling pathways and glutamatergic/GABAergic synapses (Table 5). Taken together, H3K4me3 and H3K27me3 may play distinct roles from each other primarily in cytokine and hormonal responses, and transcriptional regulation and neuronal differentiation, respectively.

Intriguingly, most of the top 20 H3K4me3 DHG expression levels were significantly altered by elevated or reduced histone modification levels, theoretically, under H3K4me3 regulation (Figure 3A). As for the 10 genes of the top H3K4me3 DHGs, the expression in LN fetuses changed >1.5-fold compared to that in HN fetuses. In contrast, seven of the top 20 H3K27me3 DHGs exhibited theoretically consistent alterations in gene expression. However, none of the expression levels significantly differed between the two fetal groups. Moreover, none of the highest H3K27me3 DHGs showed a >1.5-FC difference in expression between the two groups (Figure 3B). Thus, our results revealed that at least promoter H3K4me3 changes primarily contribute to altered gene expression in the MUN fetal calf liver. In other words, the epigenetic impact on gene expression may be regulated by the type of histone modification, at least by H3K4me3 or H3K27me3. In the MUN fetal calf liver, H3K4me3 likely plays a predominantly epigenetic role in relatively large changes in gene expression, especially in metabolically essential genes such as *APOA5*, *ANGPTL4*, *GDF15*, *FADS2*, and *PPP1R3B*.

In addition, the top 20 DMGs differed from the top 20 genes with H3K4me3 or H3K27me3 (Figure 3C). No gene showed a >1.5-FC expression difference between the two fetal groups in the top 20 DMGs. In this regard, promoter CpG methylation likely targets different genes from H3K4me3 or H3K27me3 in the MUN fetal liver. This hypothesis is supported by a similar result showing that reduced H3K4me2 and H4 acetylation, rather than DNA methylation in the promoter, caused a decrease in *CDKN2A* expression in the mammary glands of the offspring of protein-restricted rats [35]. However, even if the top 20 DMGs and H3K27me3 DHGs did not show significant changes in expression at the late gestational stage in the MUN fetal liver, it is still possible that DNA methylation or H3K27me3 may exert significant expression changes in other genes in later life stages.

### 3.2. Genes with Hyper/Hypo H3K4me3 in Hepatic Metabolisms and Function

Our results revealed a major role for H3K4me3 in regulating metabolic gene expression in the MUN fetal calf liver. The expression of *PHLDA2*, *ANGPTL4*, *MT1A*, *QRFPR*, *APOA5*, *GDF15*, *FADS2*, and *PPP1R3B* was altered >1.5-FC fold in LN fetuses compared to that in HN fetuses in response to MUN. The most striking feature of MUN-induced H3K4me3 changes is the regulation of energy metabolism genes in endocrine or cellular signaling pathways. *ANGPTL4* and *GDF15* expression was upregulated in the mouse liver under fasting conditions [36], whereas in the present study, *GDF15* but not *ANGPTL4* was upregulated, suggesting that MUN-induced adaptation was different from the response to fasting in mice. The *PPP1R3B* product functions as the catalytic subunit of protein phosphatase-1 and mediates the insulin signal by binding to dephosphorylated glycogen synthase through AKT [37] and/or the mechanistic target of rapamycin complex 1 (mTORC1) pathways [38]. It thereby plays a crucial role in hepatic glycogen deposition and thereby energy homeostasis [37,39]. Intriguingly, PPP1R3B is essentially involved in a shift in energy substrate for storage from lipid to glycogen [40], indicating that the MUN evoked switching hepatic energy usage and storage between lipid and glucose through the H3K4me3 change.

The *QRFPR* product (GPR103) is the receptor of pyroglutamylated RF-amide peptide (QRFP), a hypothalamic neuropeptide RFamide [41], and functions as a regulator of feeding activity, in addition to energy and glucose metabolism [42,43]. GPR103 is primarily expressed in the hypothalamic nuclei involved in feeding behavior control but is also abundant in gut and pancreatic islets [44]. We found that *QFRPR* transcripts were expressed in bovine liver previously [21], suggesting that GPR103 mediates modulation of energy metabolism through the peptidergic pathway in the liver. Since *QRFPR* expression is upregulated in LN fetuses, MUN likely alters fatty acid metabolism through neuroendocrinological pathways using the QRFP. Notably, the H3K4me3 of the *GDF15* promoter was also modified, accompanying altered gene expression in LN fetuses, suggesting that MUN impacted feeding behavior by suppressing neuropeptides such as GDF15 and QRFP. This neuroendocrinological metabolic alteration may be epigenetically regulated by H3K4me3 in LN fetuses.

Gene products of *APOA5* and *ANGPTL4* are abundantly produced in the liver, secreted into the plasma, and positively and negatively regulate lipoprotein lipase (LPL) activity, respectively [45], to control circulating triglycerides (TGs) [46,47]. The regulation of LPL is one of the metabolic pathways that could be affected by genes with altered H3K4me3 expression in LN fetuses in this study. Mice with *APOA5* deficiency develop hyperlipidemia with reduced post-heparin plasma LPL activity [48,49]. ANGPTL4 alone is a potent inhibitor of LPL [50]; however, its essential role is controversial owing to the variable effect of ANGPTL4 [45]. Nevertheless, in this study, MUN reduced the H3K4me3 level of the two genes, which coincidently downregulated their expression, indicating epigenetic regulation of *APOA5* and *ANGPTL4* expression by reduced H3K4me3 levels in the LUN fetal liver. Downregulation of these two genes likely reduced the regulatory effect of LPL activity and the consequent perturbation of the regulatory ability of plasma TG levels in LN fetuses. These events caused by MUN can disrupt systemic triacylglyceride (TAG) metabolism in the offspring [51].

*GDF15*, expressed in various tissues [52], is a stress-induced cytokine that plays a broad range of roles in lipid homeostasis [53,54]. Energy stress is one of the stresses that induces *GDF15* activation by the AMPK pathway [55]. Under fasting conditions, *GDF15* expression is activated by glucose homeostasis-related XBP1 in the liver to promote hepatic fatty acid β-oxidation and ketogenesis [56]. In addition, endoplasmic reticulum (ER) stress induced by tunicamycin, an inhibitor of glycoprotein synthesis in the ER, caused the direct binding of C/EBP-homologous protein (CHOP, the *DDIP3* product) in the unfolded protein response (UPR) pathway to the *GDF15* promoter, which subsequently activated *GDF15* transcription under ER stress conditions [57]. The circulating *GDF15* product likely regulates systemic energy homeostasis, including thermogenic and lipolytic genes, in brown and white adipose tissues [58,59]. These studies suggest that, in response to mitochondrial and ER stress, *GDF15* expression is activated in LN fetuses to compensate for dietary stress-induced perturbation of energy metabolism in association with alterations in lipid metabolism. We hypothesized that *GDF15* activation in MUN fetuses was induced by the UPR originating from ER stress under restricted AAs and energy substrates.

*GDF15* activation accompanied the upregulated expression of *EIF2A*, *ATF4*, *DDIT3*, and *TRIB3* in the MUN fetal liver (Figure 4). In mammalian cells, the *EIF2A* product eIF2α is phosphorylated by the *EIF2AK3* product (also known as PERK) and the *EIF2AK4* product (also known as GCN2) in response to ER stress and AA deprivation, respectively [60,61]; thus, the eIF2 kinase family regulates translation during various cellular stress conditions. The phosphorylated eIF2α then activates *ATF4*, the product of which is a major mediator of the nutrient-sensing response pathway [62]. ATF4 acts as a central transcriptional regulator of numerous genes involved in the integrated stress response (ISR) [63,64], leading to altered glucose metabolism and energy expenditure [65]. During ISR, in the eIF2α-ATF4-CHOP pathway, ATF4 activates CHOP (a *DDIT3* product) that participates in the transcriptional regulation of metabolism during hepatic ER stress [66]. CHOP likely plays a role in the adaptive functions of ISR during acute stress rather than in the induction of cell death during chronic stress conditions [67]. Upon interaction with ATF4, CHOP binds to the C/EBP-ATF element and positively regulates *TRIB3* [63]. TRIB3 is a negative feedback regulator of ATF4 [60,63]. Even when the ATF4-driven transcriptional program promotes cell death under severe stresses, TRIB3 can prolong cell survival by restricting endogenous ATF4 activity [68,69]. Thus, all factors in the eIF2α–ATF4–CHOP pathway followed by TRIB3 could be activated in the LN fetal liver for adaptation to nutritional stress, avoiding cell death even under severe stresses. This likely causes *GDF15* activation in the liver for crosstalk with cells in other organs in the systemic circulation, as suggested by a previous study [56].

There are limitations to this study, regarding statistical power and biological variability. We used four steers for each treatment, which is a small sample size for the experiments presented here. Particularly in livestock animals, individual variation in biological parameters is not small in general, and therefore the statistical significance for differences between treatments might not be appropriately detected. Nevertheless, our finding of the significant impact of MUN on H3K4me3 and gene expression does not contradict the results obtained in previous studies in sheep and other animals. Further analysis using large-scale experiments for MUN models is also required to confirm the present findings. In addition, the present results revealed a significant correlation between epigenetic H3K4me3 marks and gene expressions in MUN fetal liver, but not yet the causal effect of the epigenetic changes on gene expression in the MUN animals. The essential roles of histone modification in gene expression under MUN conditions need to be elucidated by demonstrating the direct effects of epigenetic marks by, for instance, using in vitro cell research or editing the modification.

## 4. Materials and Methods

### 4.1. Animals and Feeding

Eleven multiparous Japanese Black (JB) cows (initial BW 488 ± 9.6 kg) fed at the farm of the Western Region Agricultural Research Center (NARO) and the Iriki farm of Kagoshima University were managed and used, and fetuses were obtained as previously described [21]. Individual diets were designed for pregnant JB cows to meet 60% or 120% of the energy and other nutrient requirements based on the standard diet calculated for BW before pregnancy according to the Japanese Feeding Standard for Beef Cattle (2008 ed.) (NARO) [70]. Diet composition was designed as previously described [21]. Cows were randomly assigned to the LN or HN diet groups and fed their respective diets during gestation. Fetuses were obtained from the cows via cesarean section.

### 4.2. Sample Collection

The fetuses were injected with lidocaine (AstraZeneca, Osaka, Japan) into the jugular vein and euthanized by exsanguination at day 260 ± 8.3 of gestation. From the dissected fetal carcass, liver samples were obtained, a portion of which were frozen using liquid nitrogen for DNA preparation or soaked in RNA*later*^®^ (Thermo Fisher Scientific, Tokyo, Japan) for gene expression analysis and stored at −80 °C until used for subsequent analyses. Among the fetuses from the cows, the four with the highest BW and the four with the lowest BW were selected as HN and LN fetuses, respectively, for subsequent comparisons, including differential methylation analysis.

### 4.3. ChIP-Seq Analysis

#### 4.3.1. Chromatin Preparation and Immunoprecipitation

Genomic DNA was extracted from frozen tissues using a ChIP Reagent Kit (NIPPON GENE, Tokyo, Japan) according to the manufacturer’s protocol for preparing ChIP-seq samples. Briefly, the tissues were homogenized and crosslinked with 1% formaldehyde in PBS. The prepared chromatin was sonicated on a BIORUPTOR (SonicBio Co., Ltd., Kanagawa, Japan) using ice water. The supernatant was centrifuged at 12,000× *g* for 15 min and used for immunoprecipitation (IP). Equal volumes of each supernatant were pooled to prepare an input control.

The chromatin samples were subjected to IP using anti-H3K4me3 (Diagenode, Seraing, Belgium; cat. no.: C15410003-50, lot no.: A8034D) or anti-H3K27me3 (Merck Millipore, NJ; cat. no.: 07-449, lot no.: 2475696) antibody with Dynabeads Protein G (Thermo Fisher Scientific) in the final IP suspension, followed by overnight incubation at 4 °C. The IP chromatin was de-crosslinked, and ChIP DNAs were purified using the NucleoSpin Gel and PCR Clean-up kit (TAKARA Bio Inc., Shiga, Japan).

#### 4.3.2. Next-Generation Sequencing Analysis

Libraries were constructed from ChIP DNA samples using the ThruPLEX^®^ DNA-seq Kit (TAKARA Bio). The resulting DNAs were amplified by PCR (12 cycles) and quantified using an Agilent DNA 7500 kit (Agilent Technologies, Santa Clara, CA, USA). Sequencing was performed with paired-end reads of 36 bases for the libraries according to the concentration and data required on the Illumina NextSeq 500 platform (Illumina, San Diego, CA, USA) using the NextSeq 500/550 High Output Kit v2.5 (75 Cycles; Illumina). The raw reads obtained in FASTQ format were filtered to remove contaminated adapter sequences and low-quality reads using CLC Genomics Workbench software (ver. 23.0.2; Qiagen, Redwood, CA, USA). The read quality assessment confirmed an average PHRED score of over 20 for 99.94% and 99.72% of all reads in the H3K4me3 and H3K27me3 analyses, respectively, indicating run success. The average read number was approximately 31.4 million (H3K4me3 analysis) and 44.2 million (H3K27me3 analysis) per sample as paired-end reads. Using CLC Genomics Workbench, the obtained sequence reads were mapped to a reference genome (ARS-UCD1.2/bosTau9, April 2018). The peaks of the ChIP-seq reads were then detected on the bovine genome, and statistical analyses, such as correlation analysis, were performed using the CLC software and deep Tools (https://deeptools.readthedocs.io/, 8 May 2023) to compare the peaks of histone modification marks between the nutritional treatments and analyze differentially histone-modified regions (DHRs). Hierarchical clustering analysis (HCA) was performed for differentially histone-modified genes (DHGs) at *p* < 0.01 using cluster 3.0 (http://bonsai.hgc.jp/~mdehoon/software/cluster/manual/index.html, 25 June 2024), the results of which were visualized by Java Treeview (https://www.princeton.edu/~abarysh/treeview/, 25 June 2024).

### 4.4. Microarray Analysis

The fetuses analyzed were those with the lowest BW in the LN group (four animals) and those with the highest BW in the HN group (four animals). Total RNA samples from four fetuses in the HN and LN groups were subjected to Bovine (v2) Gene Expression 4× 44 K Microarray (Agilent). The details are described in a previous study [26]. Signals from the hybridized probes were detected using an Agilent microarray scanner (Agilent). The results were normalized by the quantile method using GeneSpring GX (Agilent).

### 4.5. Gene Ontology and Pathway Analyses

To classify the target genes according to functional annotation, GO analysis and KEGG pathway enrichment analysis (http://www.kegg.jp/, 2 June 2024) were performed on the mRNA expression profiles using the Database for Annotation, Visualization, and Integrated Discovery (DAVID) bioinformatics resources (version 6.7, http://david.abcc.ncifcrf.gov, 2 June 2024). Analysis was performed based on the distribution of DHGs or DMGs in the genome around the TSS and gene body regions (from TSS to TES). This analysis used a hypergeometric test to identify the biological metabolism and pathways significantly enriched in DHGs or DMGs compared to the entire genomic background. Statistical significance was set at *p* < 0.05.

### 4.6. Gene Expression Analysis with Quantitative PCR

Total RNAs were prepared from fetal livers, and cDNA synthesis was performed as described previously [21]. qPCR was performed using cDNA templates from the liver samples to test the expression of DHGs or DMGs. The sequences of the primers used for qPCR have been described previously [21] the ribosomal protein lateral stalk subunit P0 (*RPLP0*) was used as an internal control. A melting curve analysis was performed to confirm the specificity of the amplification reactions.

### 4.7. Comparison of Transcriptional Regulatory Potential Between Histone Methylation and DNA Methylation

To compare potential transcriptional regulation between epigenetic modifications, the top 20 differentially epigenetically modified genes for each CpG or histone methylation were tested. Data on promoter CpG-methylated genes were originally obtained from a previous study that investigated the effect of MUN on fetal liver DNA methylation [26]. It was reused to reanalyze the impact of CpG methylation in this study. DMGs were analyzed and listed using the CpG site with the greatest significant methylation level difference between LN and HN for each candidate gene. The potential impact of epigenetic factors on transcriptional alterations was first examined by determining whether each of the top 20 genes regulated by the respective epigenetic factors was regulated upward or downward by hyper- or hypo-CpG methylation, hyper- or hypo-H3K4me3 modification, and hyper- or hypo-H3K27me3 modification. Second, the frequency of genes with a >1.5-fold change (FC) in expression by MUN in the top 20 genes was counted for each epigenetic modification. Using the LN/HN ratio in epigenetic modification and gene expression as indices of epigenetic impact on altered gene expression, the richness of genes satisfying the theoretical regulation by epigenetic factors and a high FC of expression were finally evaluated as transcriptional regulatory potential. Genes not registered in the RefSeq database were not included as targets.

### 4.8. Statistical Analyses

To compare the gene expression between the LN and HN groups, BW and liver weight were analyzed using a two-sided Student’s *t*-test. The PCR data were analyzed using a one-sided Student’s *t*-test based on the trend of gene expression in a previous microarray analysis [21]. Differences were considered significant at *p* ≤ 0.05 or a trend at *p* ≤ 0.10 for the microarray and PCR results.

## 5. Conclusions

H3K4me3 in the fetal calf liver of nutrient-restricted dams was significantly upregulated in *GDF15* and *QRFPR* but downregulated in *ANGPTL4* and *APOA5*, accompanied by altered gene expression toward the upregulation or downregulation anticipated from the transcriptional activating role of the H3K4me3 mark. Regarding the impact of MUN on fetal calves, the role of H3K4me3 in gene activation is likely to have a major impact on expression changes in hepatic functional genes rather than DNA methylation. These results suggest that MUN greatly impacts hepatic ISR, systemic energy, and lipid metabolism in fetal calves, but also in later life, through altered H3K4me3 and endocrinological regulation.

## Figures and Tables

**Table 1:** Effect of maternal nutritional restriction on fetal phenotypes.

	HN (*n* = 4)		LN (*n* = 4)		*p*-Value
	Mean	SE	Mean	SE	
Age (d)	260.75	1.79	258.50	1.20	0.273
BW (kg)	32.91	0.58	24.29	1.67	0.001
Liver (g)	641.73	20.69	508.12	31.58	0.006
%BW	1.95	0.04	2.10	0.84	0.220

Data were reanalyzed for four animals for each group, using the original raw data previously reported [26]. SE, standard error.

**Table 2:** Statistical overview of differentially histone-modified regions (DHRs) between low- and high-nutrition treatments.

		DHR *	
Histone Mark	Total	Upregulated in LN	Downregulated in LN
H3K4me3	20,823	535	404
H3K27me3	35,363	1934	1909

* Count of peaks detected as DHR (*p* < 0.05) are shown. LN; low nutrition.

**Table 3:** Top 10 genes with the greatest difference in histone mark by MUN.

Histone Mark	Gene	Chr. no.	Genomic Region	LN/HN Ratio	*p*-Value
H3K4me3	*ADH4*	6	25,480,605..25,481,355	0.264	0.000003
	*USP36*	19	53,535,872..53,536,716	1.919	0.000040
	*RARB*	27	40,353,778..40,354,509	3.583	0.000047
	*LOC508098*	18	64,038,284..64,039,133	2.210	0.000060
	*LOC617302*	25	2,255,163..2,255,891	1.456	0.000069
	*MT1E*	18	24,034,824..24,035,551	1.626	0.000084
	*SLC38A4*	5	33,400,863..33,401,584	1.230	0.000100
	*ZFP36*	18	49,102,226..49,103,204	1.189	0.000127
	*PHLDA2*	29	48,689,563..48,690,308	1.695	0.000146
	*FADS1*	29	40,350,665..40,351,393	0.515	0.000164
H3K27me3	*MIC1*	23	27,912,712..27,913,557	0.407	0.000001
	*UAP1*	3	6,937,706..6,938,669	2.958	0.000003
	*LDLRAP1*	2	127,508,220..127,508,996	3.158	0.000006
	*PRTN3*	7	43,404,963..43,405,718	0.529	0.000023
	*SYT16*	10	73,935,943..73,936,680	0.468	0.000024
	*CLK2*	3	15,367,381..15,368,065	3.259	0.000027
	*HOXA11*	4	68,857,565..68,858,272	0.5	0.000065
	*TBX15*	3	24,130,717..24,131,497	0.526	0.000065
	*GFI1*	3	50,944,295..50,945,015	0.509	0.000080

**Table 4:** Biological processes and pathways associated with genes of H3K4me3 changes *.

Category	Term	*p*-Value	FE
GO_BP	GO:0006357~regulation of transcription from RNA polymerase II promoter	0.001343	2.23
	GO:0042981~regulation of apoptotic process	0.010192	5.89
	GO:0033627~cell adhesion mediated by integrin	0.021313	13.20
	GO:0016477~cell migration	0.031975	4.16
	GO:0045893~positive regulation of transcription, DNA-templated	0.034545	3.31
	GO:0071376~cellular response to corticotropin-releasing hormone stimulus	0.039141	49.87
	GO:0035767~endothelial cell chemotaxis	0.045515	42.74
	GO:0032869~cellular response to insulin stimulus	0.048273	8.47
KEGG_PATH	bta04927:Cortisol synthesis and secretion	0.000828	11.59
	bta04934:Cushing syndrome	0.003415	5.79
	bta04925:Aldosterone synthesis and secretion	0.003494	7.85
	bta05166:Human T-cell leukemia virus 1 infection	0.003907	4.55
	bta05163:Human cytomegalovirus infection	0.005195	4.29
	bta05205:Proteoglycans in cancer	0.010665	4.41
	bta05207:Chemical carcinogenesis—receptor activation	0.013152	4.19
	bta03320:PPAR signaling pathway	0.015762	7.44
	bta05200:Pathways in cancer	0.023839	2.50
	bta04928:Parathyroid hormone synthesis, secretion, and action	0.031038	5.74

* Top 10 GO biological processes (GO_BP) and KEGG pathways (KEGG_PATH) extracted at *p* < 0.05 are listed. FE, fold enrichment = (genes in list that are annotated with the term)/(total numbers of genes that are annotated with any term in the category).

**Table 5:** Biological processes and pathways associated with genes of H3K27me3 changes *.

Category	Term	*p*-Value	FE
GO_BP	GO:0006357~regulation of transcription from RNA polymerase II promoter	<0.000001	3.58
	GO:0030182~neuron differentiation	<0.000001	9.61
	GO:0009952~anterior/posterior pattern specification	<0.000001	10.47
	GO:0045944~positive regulation of transcription from RNA polymerase II promoter	<0.000001	3.21
	GO:0035115~embryonic forelimb morphogenesis	<0.000001	19.57
	GO:0048706~embryonic skeletal system development	<0.000001	18.85
	GO:0048704~embryonic skeletal system morphogenesis	<0.000001	14.14
	GO:0001764~neuron migration	0.000002	7.32
	GO:0030154~cell differentiation	0.000006	3.21
	GO:0045893~positive regulation of transcription, DNA-templated	0.000007	3.76
KEGG_PATH	bta04020:Calcium signaling pathway	0.000001	4.19
	bta05200:Pathways in cancer	0.000002	2.93
	bta04310:Wnt signaling pathway	0.000027	4.54
	bta04916:Melanogenesis	0.000040	5.99
	bta04015:Rap1 signaling pathway	0.000046	3.98
	bta04724:Glutamatergic synapse	0.000096	5.36
	bta05224:Breast cancer	0.000162	4.48
	bta05207:Chemical carcinogenesis—receptor activation	0.000204	3.67
	bta04727:GABAergic synapse	0.000679	5.37
	bta05226:Gastric cancer	0.000858	3.99

* Top 10 GO biological processes (GO_BP) and KEGG pathways (KEGG_PATH) extracted at *p* < 0.05 are listed. FE, fold enrichment = (genes in list that are annotated with the term)/(total numbers of genes that are annotated with any term in the category).

## Data Availability

The original data of annotated peaks obtained from ChIP-seq analyses are available at https://doi.org/10.5281/zenodo.14634657. Microarray data for transcriptomic analysis were deposited in the National Center for Biotechnology Information (NCBI) Gene 571 Expression Omnibus (GEO) database and are accessible through the GEO Series accession 572 number GSE176377 (http://www.ncbi.nlm.nih.gov/geo, 24 July 2025).

## Abbreviations

The following abbreviations (official gene symbol) are used in this manuscript:

Symbol	Gene name
*ABTB1*	Ankyrin Repeat And BTB Domain Containing 1
*ADH4*	Alcohol Dehydrogenase 4
*ALPL*	Alkaline Phosphatase, Biomineralization Associated
*ANGPTL4*	Angiopoietin Like 4
*APOA5*	Apolipoprotein A5
*ATF4*	Activating Transcription Factor 4
*CARD9*	Caspase Recruitment Domain Family Member 9
*CDIP1*	Cell Death Inducing P53 Target 1
*CLK2*	CDC Like Kinase 2
*COL6A1*	Collagen Type VI Alpha 1 Chain
*DDIT3*	DNA Damage Inducible Transcript 3
*DPF1*	Double PHD Fingers 1
*EIF2A*	Eukaryotic Translation Initiation Factor 2A
*EIF2AK3*	EIF2A Kinase 3/4
*EPHA5*	EPH Receptor A5
*ETV3*	ETS Variant Transcription Factor 3
*F2*	Coagulation Factor II, Thrombin
*FADS1*	Fatty Acid Desaturase 1/2
*GDF15*	Growth Differentiation Factor 15
*GFI1*	Growth Factor Independent 1 Transcriptional Repressor
*GKAP1*	G Kinase Anchoring Protein 1
*GR*	Glucocorticoid Receptor
*GNAS*	G Protein Subunit Alpha S
*G6PC*	Glucose 6 Phosphatase
*GRB10*	Growth Factor Receptor-Binding Protein 10
*HAPLN3*	Hyaluronan And Proteoglycan Link Protein 3
*HOXA11*	Homeobox A11
*IGF2*	Insulin-Like Growth Factor 2
*KBTBD6*	Kelch Repeat And BTB Domain Containing 6
*LDLRAP1*	Low-Density Lipoprotein Receptor Adaptor Protein 1
*LSP1*	Lymphocyte-Specific Protein 1
*MBOAT2*	Membrane-Bound Glycerophospholipid O-Acyltransferase 2
*METTL22*	Methyltransferase 22, Kin17 Lysine
*MIC1*	Macrophage Inhibitory Cytokine 1
*NTRK1*	Neurotrophic Receptor Tyrosine Kinase 1
*PCDHGC3*	Protocadherin Gamma Subfamily C, 3
*PDCD1*	Programmed Cell Death 1
*PHLDA2*	Pleckstrin Homology-Like Domain Family A Member 2
*PHOX2A*	Paired-Like Homeobox 2A
*PNPLA2*	Patatin-Like Domain 2, Triacylglycerol Lipase
*PODXL2*	Podocalyxin Like 2
*POR*	P450 (Cytochrome) Oxidoreductase
*PPARA*	Peroxisome Proliferator–Activated Receptor α
*PPP1R3B*	Protein Phosphatase 1 Regulatory Subunit 3B
*PROZ*	Protein Z, Vitamin K-Dependent Plasma Glycoprotein
*PRTN3*	Proteinase 3
*PTGDS*	Prostaglandin D2 Synthase
*QRFPR*	Pyroglutamylated RFamide Peptide Receptor
*RABGEF1*	RAB Guanine Nucleotide Exchange Factor 1
*RALGDS*	Ral Guanine Nucleotide Dissociation Stimulator
*RARB*	Retinoic Acid Receptor Beta
*RBFA*	Ribosome Binding Factor A
*RIPK4*	Receptor Interacting Serine/Threonine Kinase 4
*RNASEH2B*	Ribonuclease H2 Subunit B
*RPS29*	Ribosomal Protein S29
*SDC4*	Syndecan 4
*SERP1*	Stress-Associated Endoplasmic Reticulum Protein 1
*SHANK3*	SH3 And Multiple Ankyrin Repeat Domains 3
*SLC25A34*	Solute Carrier Family 25 Member 34
*SLC38A4*	Solute Carrier Family 38 Member 4
*SMARCA5*	SNF2 Related Chromatin Remodeling ATPase 5
*SYT16*	Synaptotagmin 16
*TBX15*	T-Box Transcription Factor 15
*TMEM143*	Transmembrane Protein 143
*TMEM45B*	Transmembrane Protein 45B
*TPM1*	Tropomyosin 1
*TRIB3*	Tribbles Pseudokinase 3
*TSPAN9*	Tetraspanin 9
